# Biochemical characterisation of a new thermotolerant L-asparaginase produced by *Cunninghamella echinulata* PA3S12MM isolated from the Atlantic Forest

**DOI:** 10.1007/s00203-026-04984-6

**Published:** 2026-06-06

**Authors:** Simone Von Groll, Leticia Claudia da Silva, Pedro Henrique Nascimento Souza, Wállison Justino da Silva, Thaís Duarte Bifano, José Luis da Conceição Silva, Rita de Cássia Garcia Simão, Simão, Marina Kimiko Kadowaki, Alexandre Maller

**Affiliations:** Centro de Ciências Médicas e Farmacêuticas, Universidade Estadual do Oeste do Paraná, 2069 Universitária Street, Faculdade, Cascavel, Paraná 85819-110 Brazil

**Keywords:** L-asparaginase, Filamentous fungi, Pharmaceutical industry, Food industry

## Abstract

L-asparaginase is an amidohydrolase enzyme that catalyses the hydrolysis of L-asparagine into L-aspartic acid and ammonia. This enzyme has significant biotechnological applications, functioning as an antineoplastic agent in the pharmaceutical industry and as a tool for reducing acrylamide formation in the food industry. This study aimed to identify a filamentous fungus strain capable of synthesizing a new L-asparaginase and to perform its biochemical characterisation. Among the fungal isolates screened using plate assays and submerged fermentation, *Cunninghamella echinulata* strain PA3S12MM was identified as a promising L-asparaginase producer. The highest enzymatic induction was achieved using 5% (w/v) glucose as the carbon source and 0.4% (w/v) chicken feather as an agro-industrial nitrogen source, under incubation for 120 h. The enzyme exhibited an apparent *K*_m app_ of 0.011 mmol L⁻¹ and a *V*_max app_ of 126.5 mmol L⁻¹ min⁻¹ for the hydrolysis of L-asparagine. Partial purification revealed that the enzyme displayed optimal activity at pH 7.0 and a temperature of 85 °C. In terms of substrate specificity, the enzyme demonstrated 100% relative activity toward L-asparagine and 32.9% toward L-glutamine. Enzymatic activity was enhanced by β-mercaptoethanol but inhibited by Mn²⁺, L-cysteine, and dithiothreitol (DTT). These findings suggest that *C. echinulata* PA3S12MM is a promising candidate for large-scale biotechnological production of a new fungal L-asparaginase, with biochemical indicators of potential for applications in both pharmaceutical and food industries.

## Introduction

L-asparaginase is an amidohydrolase enzyme (E.C. 3.5.1.1) that catalyses the hydrolysis of the amino acid L-asparagine into L-aspartic acid and ammonia (Shrivastava et al. 2016). This enzyme has broad industrial applications. In the food industry, L-asparaginase is employed to mitigate the formation of acrylamide, a potentially toxic and carcinogenic compound generated during the thermal processing of carbohydrate- and asparagine-rich foods such as fried potatoes, bread, and biscuits (Jia et al. 2021). Furthermore, L-asparaginase is utilised as a therapeutic agent in the treatment of haematological malignancies, particularly acute lymphoblastic leukaemia (ALL) and Hodgkin’s lymphoma (Cachumba et al. 2016).

L-asparaginase is produced by a wide range of organisms, including bacteria, fungi, algae, and plants. Among fungal sources, *Aspergillus niger* and *Aspergillus oryzae* are the most extensively utilised species in the food industry and are classified as Generally Recognized as Safe (GRAS) by the United States Food and Drug Administration (FDA) (da Cunha et al. 2019). In the pharmaceutical sector, L-asparaginase is primarily derived from two bacterial strains, *Escherichia coli* and *Erwinia chrysanthemi* (Muneer et al. 2020). However, L-asparaginase of prokaryotic origin has been associated with adverse effects, including central nervous system thrombosis and pancreatitis, thereby complicating treatment and increasing the risk of relapse in paediatric acute lymphoblastic leukaemia (ALL) (Baruchel et al. 2020). Given their high enzymatic productivity and resilience to environmental fluctuations, filamentous fungi have been investigated as alternative sources of L-asparaginase, offering the potential for reduced toxicity (Muneer et al. 2020).

The genus Cunninghamella encompasses species of considerable importance in both medical mycology and biotechnological applications. These filamentous fungi are commonly isolated from soil and plant material, particularly in Mediterranean and subtropical regions (Asha and Vidyavathi 2009). Moreover, these organisms possess the ability to metabolize a wide range of pharmaceuticals through enzymatic pathways that closely resemble those found in mammalian systems (Ma et al. 2007). With respect to the enzymatic potential of *Cunninghamella echinulata*, previous studies have reported the production of amylase (Cavalheiro et al. 2023) and invertase (Rasbold et al. 2022). More recently, L-asparaginase has been identified in this species through a systematic screening approach aimed at isolating strains capable of synthesizing this enzyme (Ramos et al. 2024).

Although the production of L-asparaginase by various microbial species has been extensively studied, no reports to date have detailed the biochemical characterisation of this enzyme in *C. echinulata*. Therefore, the present study aimed to investigate the production of L-asparaginase by fungi isolated from the Atlantic Forest and to characterize the biochemical properties of the enzyme synthesized by *C. echinulata* PA3S12MM.

## Materials and methods

### Maintenance of strains

The experiments were conducted using five filamentous fungi previously collected from a fragment of the Atlantic Forest, located in the municipality of Nova Aurora, Paraná, Brazil, at coordinates 24° 32’ 00’’ S and 53° 15’ 10’’ W, at an altitude of 520 m above sea level (Rasbold et al. 2022). The fungal strains are preserved in the Fungal Collection of the Laboratory of Microbial Biochemistry at the State University of Western Paraná, Cascavel, Brazil. Strain maintenance was performed in sterile test tubes containing 15 mL of potato dextrose agar (PDA) medium, followed by incubation at 28 °C for eight days.

### Screening of L-asparaginase producing fungi

The screening of microorganisms capable of producing L-asparaginase was performed using a semi-quantitative evaluation on Petri dishes and submerged fermentation. The semi-quantitative assessment on Petri dishes was conducted using modified Czapek Dox medium (Gulati et al. 1997), composed of glucose (0.2%), L-asparagine (1%), NH₄NO₃ (0.2%), KH₂PO₄ (0.152%), KCl (0.052%), MgSO₄·7 H₂O (0.052%), ZnSO₄·7 H₂O (0.0001%), CuSO₄·7 H₂O (0.0001%), and FeSO₄·7 H₂O (0.0001%), supplemented with 0.0012% phenol red as an indicator and 2% agar. Fungal spores were inoculated at the centre of the plate and incubated at 30 °C for five days. A negative control plate was prepared without phenol red and L-asparagine. The presence of a red halo surrounding the fungal colony indicated L-asparaginase activity.

The diameters of the hydrolysis halos and fungal colonies were measured using a calliper at 24 h, and the results were expressed as the enzymatic index (EI), calculated according to Eq. 1 (Gulati et al. 1997):

1 $$\:\mathrm{E}\mathrm{I}:\frac{\mathrm{H}\mathrm{a}\mathrm{l}\mathrm{o}\:\mathrm{D}\mathrm{i}\mathrm{a}\mathrm{m}\mathrm{e}\mathrm{t}\mathrm{e}\mathrm{r}}{\mathrm{C}\mathrm{o}\mathrm{l}\mathrm{o}\mathrm{n}\mathrm{y}\:\mathrm{D}\mathrm{i}\mathrm{a}\mathrm{m}\mathrm{e}\mathrm{t}\mathrm{e}\mathrm{r}}$$

Subsequently, L-asparaginase production under submerged fermentation conditions was assessed by inoculating 1.5 mL of a 10⁵ spores mL⁻¹ suspension (0.8% NaCl and 0.5% Tween 80) into a 125 mL Erlenmeyer flask containing 25 mL of liquid modified Czapek Dox medium (Gulati et al. 1997), in which L-asparagine was substituted with proline. The medium was adjusted to pH 8.5 and incubated at 28 °C for 120 h under static conditions. Aliquots were collected every 24 h for the determination of enzymatic activity (Imada et al. 1973) and protein concentration (Bradford 1976).

### Production of L-asparaginase by ***C. echinulata*** PA3S12MM

For L-asparaginase production, a spore suspension (10^5^ spores mL^− 1^) was inoculated into 125 mL Erlenmeyer flasks containing 25 mL of Czapek-Dox medium (Gulati et al. 1997), supplemented with 5% (w/v) glucose as carbon source, 0.5% (w/v) asparagine, and 0.4% (w/v) chicken feathers as nitrogen source. The flasks were incubated at 28 °C for 120 h under static conditions. Following incubation, the cultures were filtered using Whatman No. 1 filter paper to obtain a cell-free crude enzymatic extract.

### Enzyme activity measurement and protein quantification

L-asparaginase activity was assessed using a modified protocol based on the method described by Imada et al. (1973). The reaction mixture consisted of 100 µL of a 0.1 mol L⁻¹ L-asparagine solution, 160 µL of 50 mmol L⁻¹ Tris-HCl buffer (pH 8.0), and 40 µL of the enzyme extract, incubated at 37 °C for 30 min. The reaction was terminated by the addition of 100 µL of 1.5 mol L⁻¹ trichloroacetic acid. Subsequently, a 25 µL aliquot of the reaction mixture was transferred to a microplate containing 200 µL of distilled water and 25 µL of Nessler’s reagent (Merck^®^), followed by incubation at 28 °C for 15 min. Absorbance was measured at 450 nm using a spectrophotometer. Enzyme activity (U mL⁻¹) was defined as the amount of enzyme required to release 1 µmol of ammonia per minute under the assay conditions.

Protein quantification was performed using the Bradford method (Bradford 1976), with bovine serum albumin (BSA) as the standard.

### Effect of carbon and nitrogen sources on L-asparaginase production

The influence of different carbon sources on L-asparaginase production by *C. echinulata* PA3S12MM was assessed by supplementing modified Czapek medium with 1% (w/v) agro-industrial residues, including sugarcane bagasse, banana peel, orange peel, potato peel, cassava peel, wheat bran, soybean flour, soybean straw, sorghum bran, *Ora-pro-nóbis* leaves (*Pereskia aculeata* Miller), lactose, and glucose. The effect of nitrogen sources was evaluated by supplementing the modified Czapek medium with 0.4% (w/v) of various nitrogenous substrates, including asparagus, peptone, tryptone, casein, meat extract, chicken feathers, and a control condition with no additional nitrogen source. All cultures were incubated at 28 °C for 144 h under static conditions.

### Purification of L-asparaginase from *C.echinulata* PA3S12MM

The crude enzymatic extract was lyophilized and resuspended in 900 µL of 0.02 mol L⁻¹ sodium citrate buffer (pH 5.0). The suspension was then applied to a Sephacryl S-100 h gel filtration column pre-equilibrated with the same buffer. All purification steps were performed at 4 °C. Fractions of 1 mL were collected and subsequently analysed for enzymatic activity and protein concentration. Fractions exhibiting L-asparaginase activity were pooled and filtered through a 0.22 μm microporous membrane for further analysis using a High-Performance Liquid Chromatography (HPLC) system (Shimadzu LC-20 model). The purified sample was then subjected to an additional purification step using a Sephacryl S-200 h gel filtration column (GE Healthcare), employing a mobile phase composed of 50 mmol L⁻¹ sodium phosphate buffer (pH 7.0) containing 150 mmol L⁻¹ NaCl. The chromatographic process was conducted at a flow rate of 0.8 mL min⁻¹, with a sample injection volume of 100 µL.

### Purity analysis

The enzyme sample was analysed by sodium dodecyl sulphate-polyacrylamide gel electrophoresis (SDS-PAGE) under denaturing conditions, following the protocol described by Laemmli (1970). A 6% stacking gel and a 10% running gel were utilised for protein separation. Electrophoresis was conducted at 120 V and 20 mA at room temperature. The molecular weight marker employed was the PageRuler^™^ Plus Prestained Protein Ladder (Thermo Scientific^™^), with a molecular weight range of 10–250 kDa. Following electrophoresis, the gel was stained using a silver nitrate staining method to visualize protein bands.

### Effect of temperature and pH on enzyme activity and stability

The effect of temperature on L-asparaginase activity was evaluated by measuring enzymatic activity across a temperature range of 30 to 100 °C. Thermal stability was assessed by incubating the enzyme in the absence of substrate at 85, 90, and 95 °C for 180 min, followed by the determination of residual enzymatic activity at the enzyme’s optimal temperature (Imada et al. 1973).

The influence of pH on L-asparaginase activity was examined by performing the enzymatic reaction in 0.1 mol L⁻¹ citrate-phosphate buffer (McIlvaine 1921) over a pH range of 3.0 to 8.0. Enzyme stability at different pH values was assessed by incubating the enzyme, without substrate, in the same buffer and pH conditions at 4 °C for 24 h. After this incubation period, residual enzymatic activity (Imada et al. 1973) was measured at the enzyme’s optimal temperature and pH.

### Effect of ions and other compounds on L-asparaginase activity

The influence of various metal ions and chemical compounds on L-asparaginase activity was assessed by incorporating the respective substances into a reaction mixture containing 0.1 mol L⁻¹ L-asparagine as the substrate, prepared in 0.1 mol L⁻¹ citrate-phosphate buffer (pH 7.0). The tested metal ions included BaCl₂, CuCl₂, CoCl₂, FeCl₂, KCl, MgCl₂, MnCl₂, NaCl, AgNO₃, K₂SO₄, MnSO₄, NH₄NO₃, and HgCl₂. Additionally, the effects of L-cysteine, ethylenediaminetetraacetic acid (EDTA), dithiothreitol (DTT), and 2-mercaptoethanol were evaluated. Enzymatic activity was measured following the addition of these compounds (Imada et al. 1973), with final concentrations of 5 mmol L⁻¹ and 10 mmol L⁻¹ tested for each ion.

### Kinetic studies

The apparent kinetic parameters (*K*_m app_, *V*_max app_, and *K*_cat app_) (Michaelis and Menten 1913) for L-asparaginase were determined through enzymatic assays using L-asparagine as the substrate at concentrations ranging from 0.02 to 0.3 mmol L⁻¹, under optimal temperature and pH conditions.

### Substrate specificity

The substrate specificity of L-asparaginase was evaluated by conducting enzymatic assays using L-asparagine, L-proline, L-glutamine, and glycine, each at a concentration of 0.1 mol L⁻¹. Enzymatic activity was subsequently quantified for each substrate.

### Statistical analysis

Data analysis was performed using one-way analysis of variance (ANOVA), and multiple comparisons were conducted using Tukey’s test, with a significance threshold of *p* ≤ 0.05. Statistical analyses were carried out using GraphPad Prism (version 10.3.1). Kinetic constants were determined using Enzyplot software, and graphical data representations were generated using Origin 6.1 software.

## Results

### Screening of L-asparaginase producing fungi

**Fig. 1:**
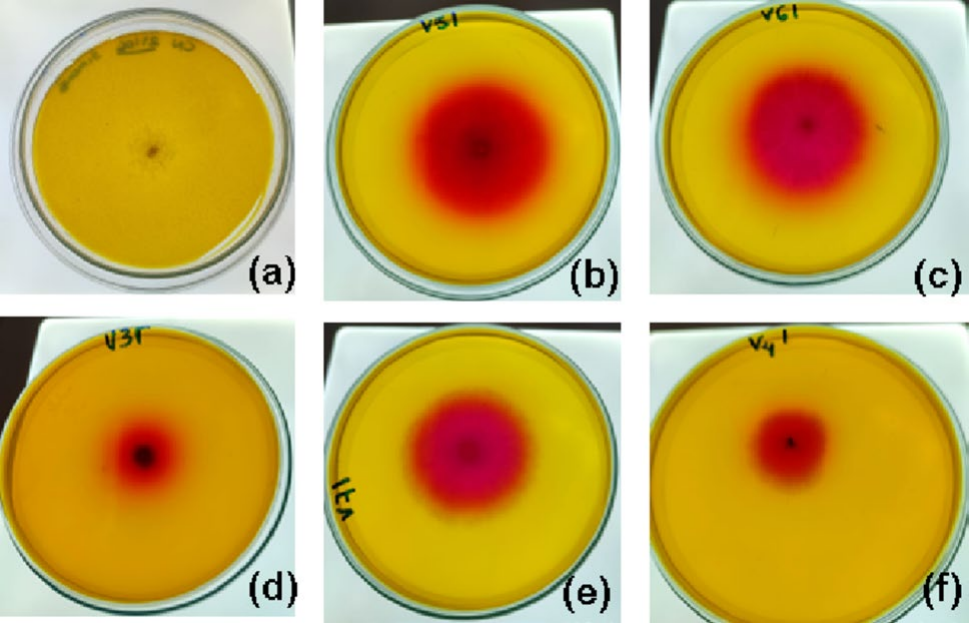
Screening of fungi isolated from a fragment of the Atlantic Forest for L-asparaginase production on solid medium. **a** Negative control; **b** PA2S7MM; **c** *C. echinulata* PA3S12MM; **d** PA2S4MY; **e** PA3S17MC; **f** PA1S2MV

**Fig. 2:**
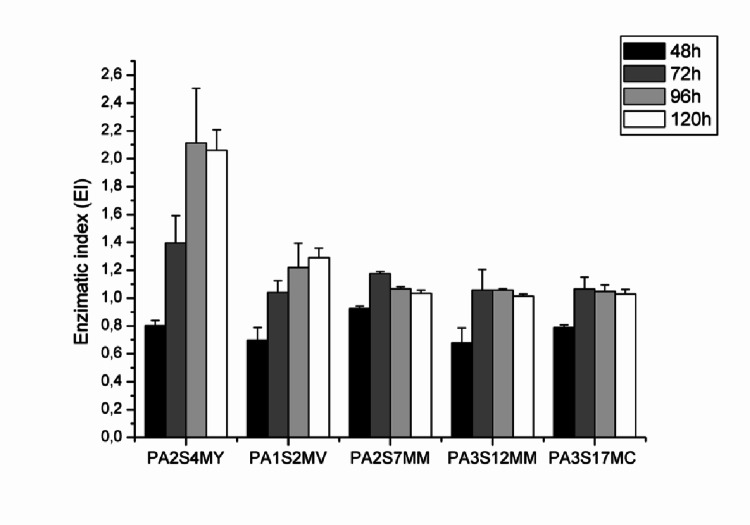
Enzyme index of fungi screened for L-asparaginase production on solid medium

Five fungal isolates obtained from a fragment of the Atlantic Forest exhibited L-asparaginase production in a semi-qualitative plate assay after 48 h of incubation (Fig. 1). The presence of L-asparaginase activity was indicated by the formation of a red halo around the fungal colonies, resulting from the hydrolysis of asparagine into aspartic acid and ammonia, which caused a colour shift in the phenol red indicator from yellow to pink. The enzymatic index values, as shown in Fig. 2, ranged from 0.68 to 2.1, confirming L-asparaginase production in all tested microorganisms.

Furthermore, all five fungal strains produced L-asparaginase in liquid medium during 120 h of submerged fermentation. Enzymatic activity levels ranged from 4.79 U mL^− 1^ for *C. echinulata* PA3S12MM to 1.79 U mL^− 1^ for PA2S7MM under submerged culture conditions.

**Fig. 3:**
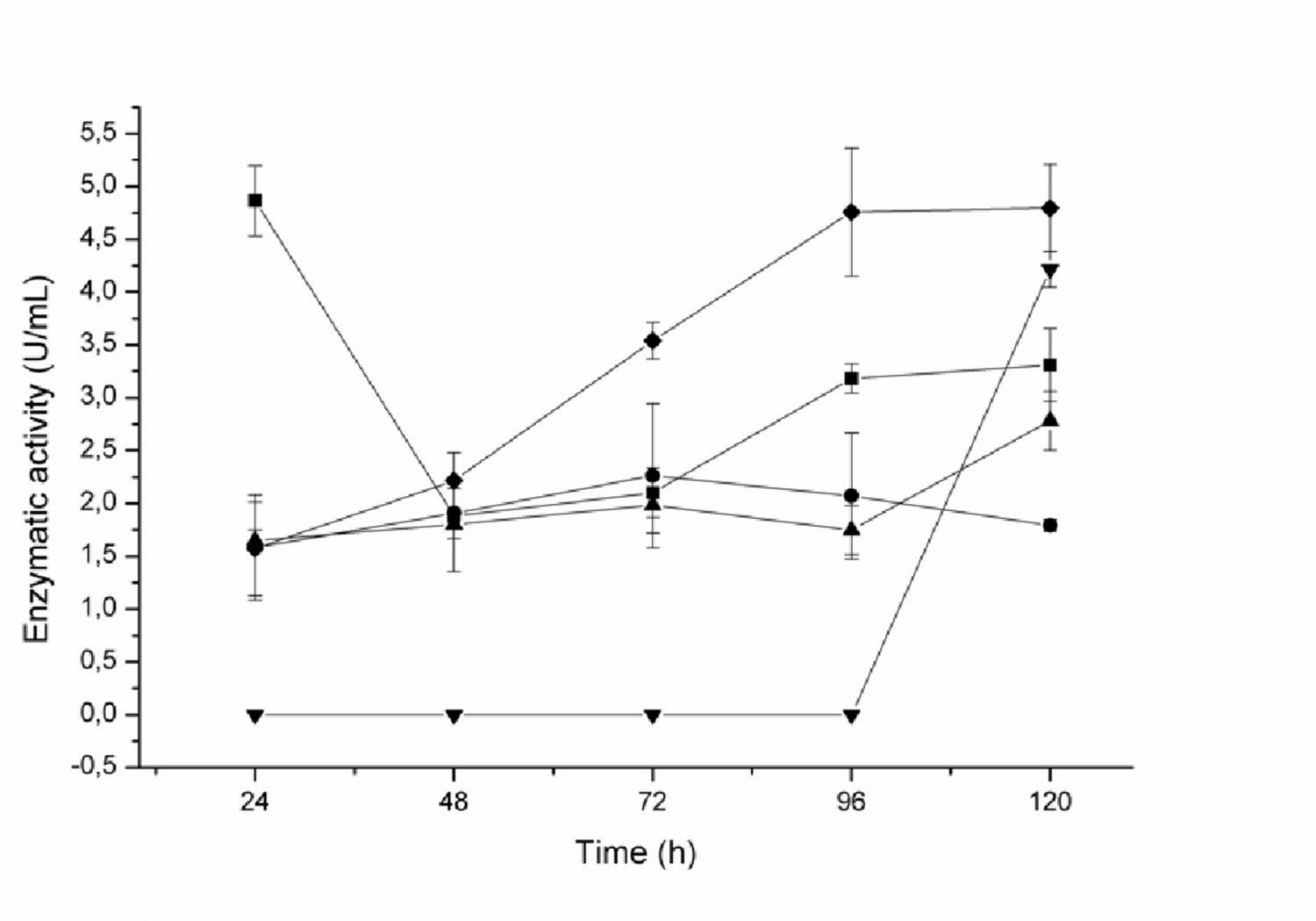
Screening of fungi isolated from a fragment of the Atlantic Forest for L-asparaginase production under submerged fermentation conditions. (■) PA1S2MV, (⦁) PA2S7MM, (▲) PA3S17MC, (▼) PA2S4MY, (♦) *C. echinulata* PA3S12MM

In this study, *C. echinulata* PA3S12MM was selected for further investigation of L-asparaginase production in submerged fermentation due to the absence of literature data on enzyme production by this strain (Fig. 3).

### Influence of carbon and nitrogen sources on L-asparaginase production

**Fig. 4:**
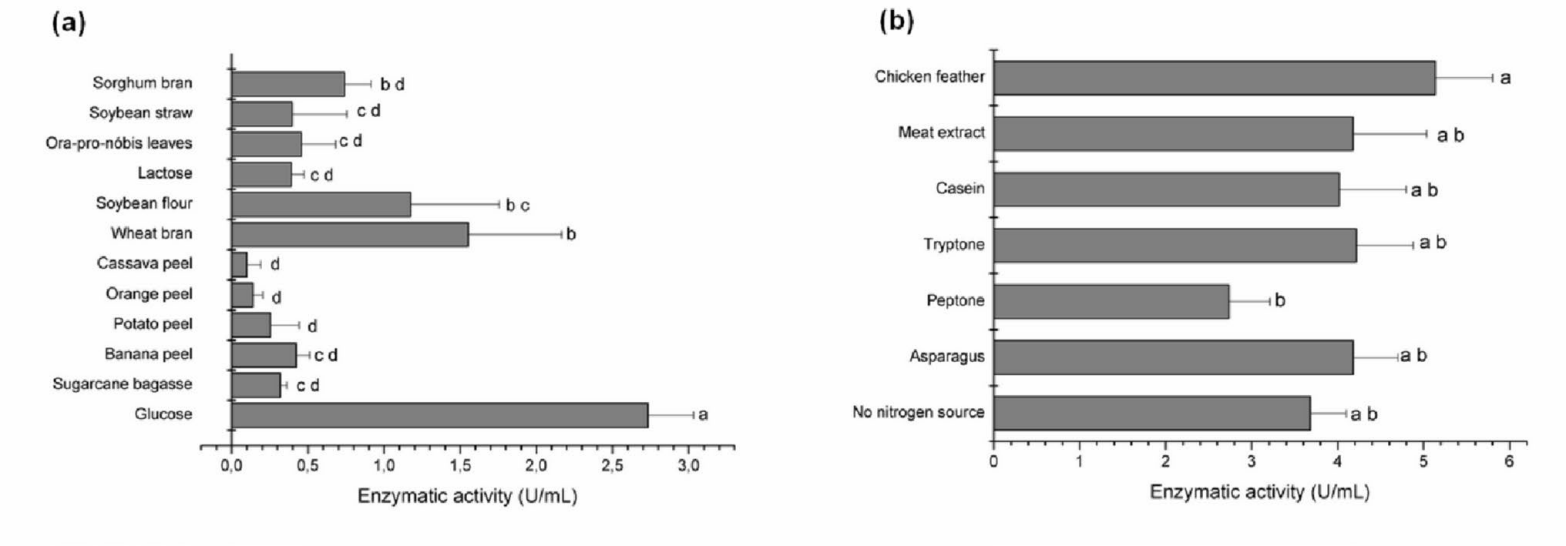
Optimisation of culture conditions for L-asparaginase production by *C. echinulata* PA3S12MM. **a** Effect of carbon sources; **b** Effect of nitrogen sources. Data are expressed as means ± standard deviation from three replicates. Different letters (a-d) indicate significant differences according to Tukey´s test (*p* ≤ 0.05)

Among the tested carbon sources, 1% glucose resulted in the highest L-asparaginase activity (3.5 U mL⁻¹), followed by agro-industrial residues, including glucose and soy flour (2.0 U mL⁻¹), wheat bran (1.55 U mL⁻¹), and soy flour (1.17 U mL⁻¹) (Fig. 4a). With respect to nitrogen sources, chicken feathers, an agro-industrial residue, induced the highest enzymatic activity (5.14 U mL⁻¹), followed by asparagus, tryptone, and meat extract, each yielding 4.2 U mL⁻¹ (Fig. 4b).

### Purification of L-asparaginase

**Fig. 5:**
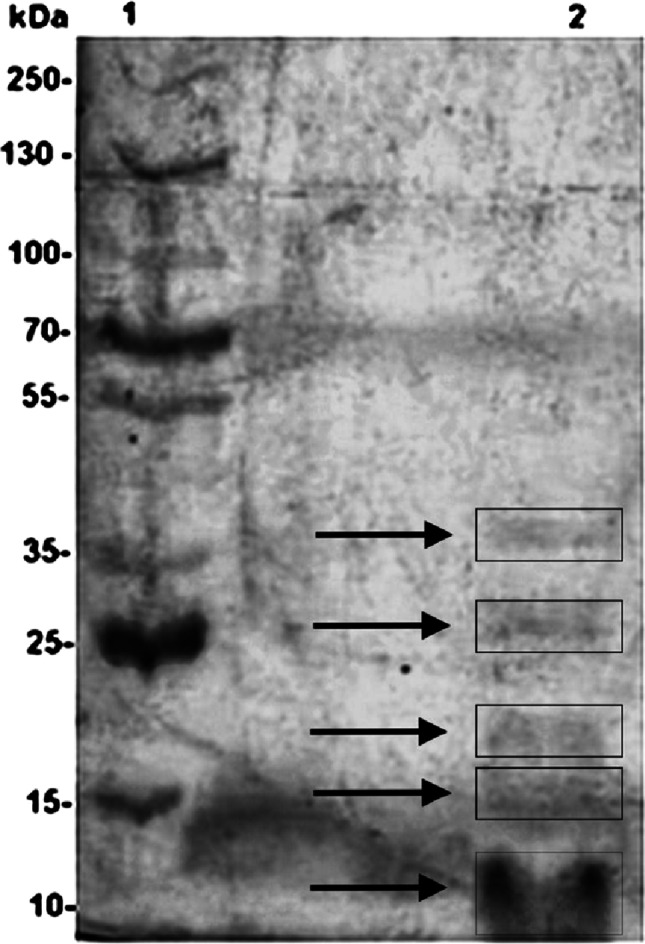
SDS-PAGE analysis of L-asparaginase from *C. echinulata* PA3S12MM. (1) Molecular weight marker (10–250 kDa); (2) Partially purified L-asparaginase. Electrophoresis was performed using a 6% stacking gel and a 10% running gel at 120 V and 20 mA at room temperature. The gel was stained with silver nitrate

The crude enzymatic extract was subjected to size-exclusion chromatography using a Sephacryl S-100 h column, yielding approximately 20 mL of fractions exhibiting enzymatic activity, with a specific activity of 13.9 U·mg⁻¹. The active fractions were subsequently applied to a Sephacryl S-200 h column (GE Healthcare), where enzymatic activity was detected in 4.3 mL of collected fractions, presenting a specific activity of 89.3 U·mg⁻¹. The purification process resulted in a purification factor of 10.4, with a final enzyme recovery of only 0.2% (Table 1). SDS-PAGE analysis revealed the presence of four protein bands with molecular weights below 35 kDa in the purified sample (Fig. 5), indicating that the enzyme was only partially purified.

**Table 1:** Partial purification of L-asparaginase produced by *C. echinulata* PA3S12MM

Purification steps	Volume (mL)	Total activity (U)	Total protein (mg)	Specific activity (U mg^− 1^)	Yield (%)	Purification factor (x)
Crude extract	412	1194.8	138.8	8.6	100.0	1.0
Sephacryl S-100 h	20	324.6	23.4	13.9	27.0	1.6
HPLC	4.3	11.4	0.13	89.3	0.2	10.4

### Effect of temperature and pH on enzyme activity and stability

**Fig. 6:**
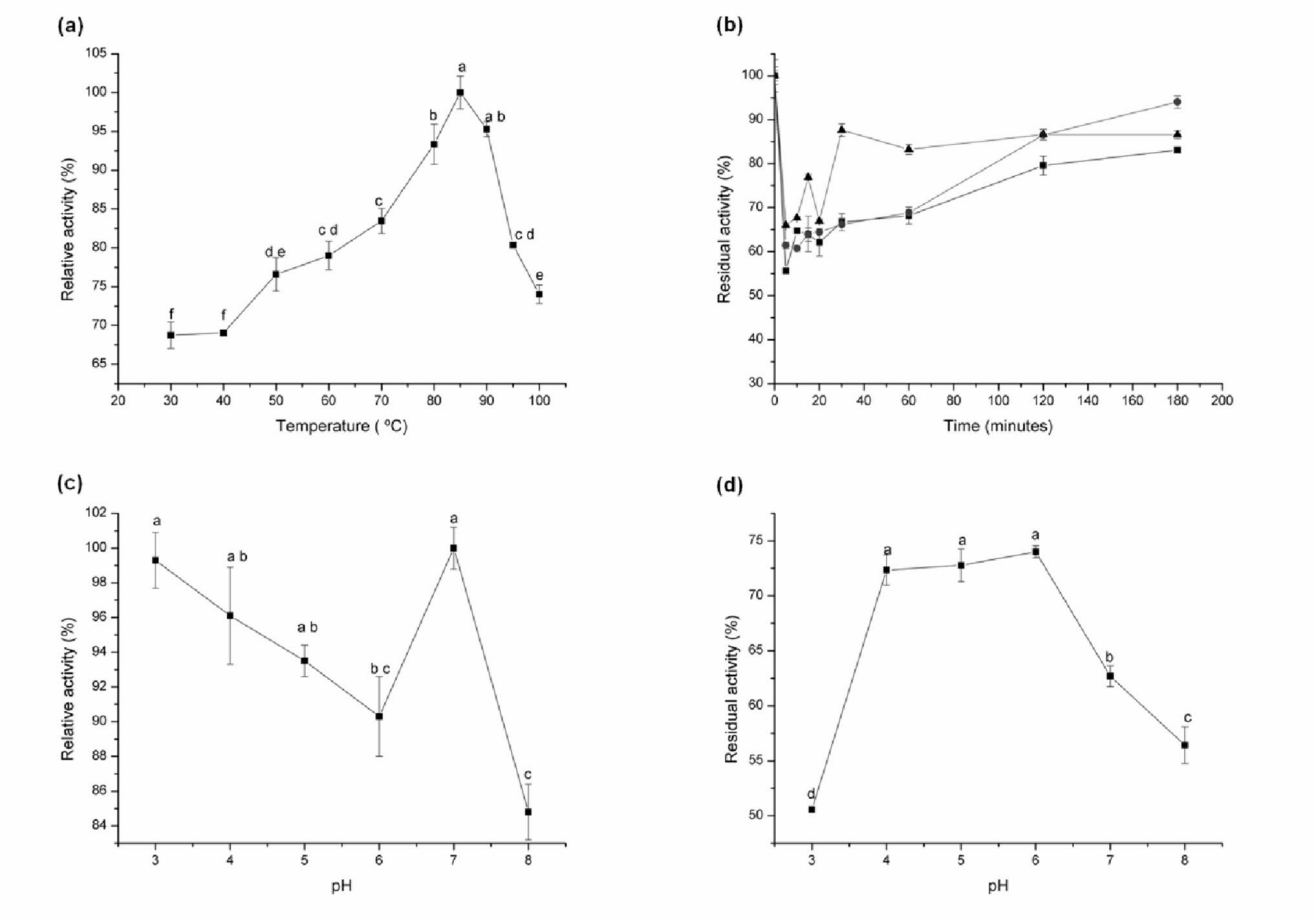
Effect of temperature and pH on L-asparaginase activity and stability. **a** Optimum temperature; **b** Thermal stability at (■) 85 °C, (⦁) 90 °C, and (▲) 95 °C; **c** Optimum pH; **d** pH stability. Data are expressed as means ± standard deviation from three replicates. Different letters (a–f) indicate significant differences according to Tukey´s test (*p* ≤ 0.05)

The optimum temperature for L-asparaginase activity was determined to be 85 °C (Fig. 6a). Thermal stability assays demonstrated that the enzyme remained stable across all tested temperatures (85, 90, and 95 °C). Notably, at 95 °C, the enzyme exhibited thermal activation after 30 min of incubation (Fig. 6b). After 180 min of incubation at 90 °C, L-asparaginase retained approximately 94% of its initial activity.

L-asparaginase from *C. echinulata* PA3S12MM displayed optimal activity at pH 7.0, with 84.8% relative activity observed at pH 8.0 (Fig. 6c). At more acidic pH values, the enzyme exhibited relative activity ranging from 99.3% to 93.5%.

Regarding enzymatic stability, L-asparaginase maintained its activity within the pH range of 4.0–6.0 after 24 h of incubation, retaining 74% activity at pH 6.0. However, at pH 3.0, the enzyme retained only 50% of its residual activity (Fig. 6d).

### Effect of ions and other compounds on L-asparaginase activity

The influence of various compounds on L-asparaginase activity was evaluated at 5 mmol L⁻¹ and 10 mmol L⁻¹ concentrations under optimal reaction conditions. The reducing agent β-mercaptoethanol significantly enhanced enzymatic activity, resulting in a 72.1% increase.

Conversely, ethylenediaminetetraacetic acid (EDTA) at 10 mmol L⁻¹ reduced enzymatic activity by 51.4%, suggesting that L-asparaginase may function as a metalloprotein.

As shown in Table 2, dithiothreitol (DTT) at 10 mmol L⁻¹ led to an 87.8% reduction in enzymatic activity. Additionally, MnSO₄ at 10 mmol L⁻¹ completely inhibited the activity of the purified enzyme. Among the metal ions tested, FeCl₂ and KCl exhibited a negative effect on enzymatic activity.

**Table 2:** The effect of various compounds on L-asparaginase activity

Compound	Relative activity (%)	
	5 mmol L^− 1^	10 mmol L^− 1^
Control	100	100
CoCl_2_	54.6 ± 0.02	46.6 ± 0.06
BaCl_2_	55.3 ± 0.04	68.9 ± 0.03
CuCl_2_	64.8 ± 0.03	88.8 ± 0.06
DTT	36.6 ± 0.01	12.2 ± 0.05
EDTA	79.7 ± 0.07	48.6 ± 0.05
FeCl_2_	35.8 ± 0.02	60.7 ± 0.07
NaCl	59.8 ± 0.03	75.1 ± 0.09
MgCl_2_	45.6 ± 0.01	49.2 ± 0.05
AgNO_3_	45.6 ± 0.05	64.2 ± 0.05
MnCl_2_	22.2 ± 0	20.3 ± 0.26
KCl	0.8 ± 0.01	3.6 ± 0.02
L-cysteine	23.2 ± 0.06	20.1 ± 0.14
K_**2**_SO_4_	59.3 ± 0.05	69.3 ± 0.08
MnSO_4_	30.2 ± 0.12	ND*
Beta-mercaptoethanol	166 ± 0.06	172.1 ± 0.06
NH_4_NO_3_	49 ± 0.04	21.5 ± 0.05
HgCl_2_	50 ± 0.04	30.4 ± 0.08

*ND : Not detected

### Determination of kinetic constants and substrate specificity

**Fig. 7:**
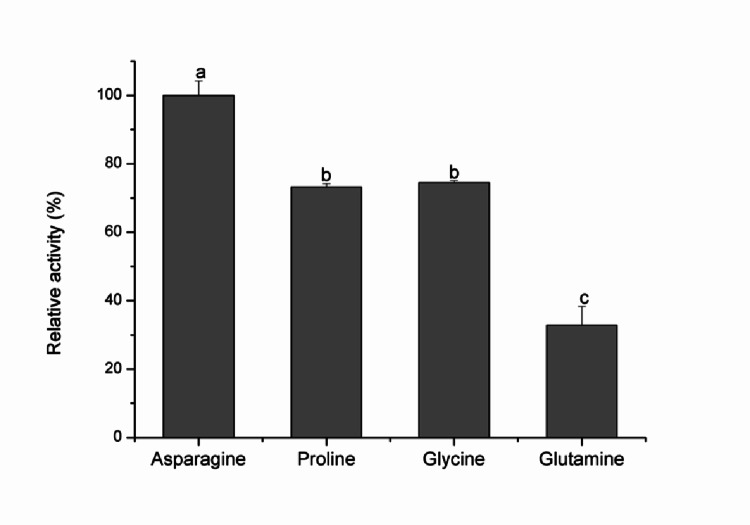
Substrate specificity of L-asparaginase from *C. echinulata* PA3S12MM. Different letters indicate significant differences according to Tukey´s test (*p* ≤ 0.05)

L-asparaginase exhibited apparent *K*_m app_ and *V*_max app_ values of 0.011 mmol L^− 1^ and 126.5 mmol L^− 1^ min^− 1^, respectively, for the substrate L-asparagine. The enzyme specificity was assessed using various amino acids, as illustrated in Fig. 7. L-asparaginase demonstrated the highest specificity for L-asparagine, while exhibiting only 32.9% relative activity toward L-glutamine.

## Discussion

The primary objective of this study was to identify a promising filamentous fungus for L-asparaginase production. All tested strains exhibited the ability to synthesize this enzyme. However, *C. echinulata* PA3S12MM was identified as the most efficient producer under submerged fermentation conditions. To date, only one study has reported L-asparaginase production by this species, specifically the DSM1905 strain. In that study, the enzyme production was evaluated using three different pneumatically agitated bioreactors for comparative analysis (Ramos et al. 2024). This highlights the novelty of the present work, particularly in relation to the enzymatic potential of *C. echinulata* isolated from Atlantic Forest environments.

Microorganisms are recognized as the primary sources of natural bioactive compounds with significant applications in agriculture, medicine, and the pharmaceutical industry (Chow and Ting 2015). Among these, filamentous fungi have been extensively explored due to their metabolic diversity and capacity to produce extracellular enzymes with industrial relevance.

Recent studies have demonstrated that several fungal genera are capable of producing L-asparaginase, with the majority belonging to the genus Fusarium (Wei et al. 2024). However, the production levels and biochemical characteristics of the enzyme vary significantly depending on the species and cultivation conditions (Shakambari et al. 2018).

In this context, our study demonstrated that 1% glucose is the ideal carbon source to maximize enzyme production. Similarly, the literature reports that the highest L-asparaginase production by *Fusarium equiseti* AHMF4 was achieved in culture media supplemented with 1% glucose (El-Gendy et al. 2021). In contrast, *Penicillium crustosum* exhibited maximum enzyme production when cultivated with 1.69% sucrose as the primary carbon source (Khalil et al. 2021).

Furthermore, our study investigated various nitrogen sources, including agro-industrial residues, such as chicken feathers. Submerged fermentation cultures supplemented with chicken feathers yielded the highest enzymatic production. A comparable study reported that 0.19% peptone resulted in 16.53 U mL^− 1^ of L-asparaginase activity in *P. crustosum* cultures (Khalil et al. 2021).

Extracellular L-asparaginase was partially purified using size-exclusion chromatography and high-performance liquid chromatography (HPLC), yielding a recovery rate of 0.2%. The substantial loss of enzymatic activity during chromatographic purification limited further purification steps, ultimately resulting in a semi-purified enzyme with a low final yield. These findings highlight the necessity for process optimisation to facilitate the enzyme’s application on an industrial scale.

A promising approach to enhance enzyme recovery and improve overall purification efficiency would involve multiple purification cycles combined with lyophilization to concentrate the enzyme. Additionally, further studies on enzyme immobilization could offer a viable strategy for optimizing its large-scale application. Comparative studies in the literature report recovery rates of 34.9% for intracellular L-asparaginase from *Chaetomium* sp. (Arumugam and Thangavelu 2022) and 39.89% for the enzyme from *Fusarium foetens* (Parashiva et al. 2023). Conversely, L-asparaginase isolated from *Aspergillus oryzae* spp. exhibited a much lower recovery rate of 0.30% (da Cunha et al. 2022).

Our assays evaluating the effect of temperature on the activity of L-asparaginase from *C. echinulata* PA3S12MM yielded results comparable to those reported for the recombinant enzyme from the hyper thermophilic archaeon *Thermococcus kodakarensis* KOD1. In that study, the authors investigated the cloning, expression, and biochemical characterisation of L-asparaginase in *Escherichia coli* BLR (DE3), identifying the enzyme as hyper thermostable, with an optimal temperature of 90 °C (Hong et al. 2014). The high optimal temperature observed for L-asparaginase from *C. echinulata* PA3S12MM may be attributed to the presence of cysteine residues in its primary structure, a characteristic frequently observed in proteins from hyper thermophilic organisms. These residues may contribute to structural stabilization through the formation of disulfide bridges and metal coordination, or they may be situated in regions that are shielded from solvent exposure (Dumina and Zhgun 2023). These structural attributes are likely key determinants of the enzyme’s enhanced thermal stability and may play a role in defining its optimal temperature.

The thermal stability assessment of L-asparaginase from *C. echinulata* PA3S12MM revealed that the enzyme retained 86.6% of its activity after 3 h of incubation at 95 °C. This finding is consistent with previous reports on L-asparaginase from *Thermococcus kodakarensis*, which maintained 90% residual activity at 90 °C for up to 32 h (Hong et al. 2014).

Regarding pH, the enzyme exhibited optimal activity under slightly acidic to neutral conditions, which is in agreement with previous studies involving fungal L-asparaginases, such as those produced by *Mucor hiemalis* (Thakur et al. 2014) and *Penicillium digitatum* (Shrivastava et al. 2012). Similar pH profiles have also been reported for *Chaetomium *sp. (Arumugam et al. 2021), suggesting that this characteristic is relatively conserved among fungal enzymes.

Metals play a crucial role in maintaining the structural integrity of many proteins, contributing to enzymatic stability and enhancing substrate-binding efficiency. As a result, enzymatic activity can be modulated by different metal ions, which may function as activators or inhibitors (Kim et al. 2020). In this study, the effect of various compounds on the activity of L-asparaginase from *C. echinulata* PA3S12MM was evaluated. The results demonstrated that the reducing agent β-mercaptoethanol significantly enhanced enzymatic activity. This finding is consistent with reports on L-asparaginase from *Aspergillus oryzae* IOC 3999, where enzymatic activity increased by 185.8% in the presence of β-mercaptoethanol (da Cunha et al. 2022). The observed enhancement in enzyme activity can be attributed to the cleavage of disulfide bonds near the enzyme’s active site by β-mercaptoethanol. This reaction likely exposes highly reactive thiol groups, thereby increasing catalytic efficiency by reducing conformational rigidity and enhancing substrate accessibility (Trivedi et al. 2009).

L-asparaginase from *C. echinulata* PA3S12MM exhibited the highest enzymatic activity with L-asparagine (248.2 U mL⁻¹) compared to the other amino acids tested. Its high affinity for L-asparagine and low activity towards L-glutamine (32.9%) suggest its potential applicability in the food industry, particularly in acrylamide mitigation. However, the enzyme retained approximately 75% of its activity when tested with proline and glycine. Similar findings have been reported for fungal-derived L-asparaginases, including those from *Aspergillus oryzae* spp. (da Cunha et al. 2022) and *Penicillium crustosum* (Khalil et al. 2021), which also demonstrated higher affinity for L-asparagine.

The *K*_m app_ of L-asparaginase from *C. echinulata* PA3S12MM was lower than *K*_m_ reported for the enzymes from *Aspergillus niger* (*K*_m_ = 0.8141 mM) (Vala et a. 2018) and *Chaetomium *sp. (*K*_m_ = 257.1 mM) (Arumugam et al. 2021) and was comparable to that of *Penicillium digitatum* (*K*_m_ = 1 × 10⁻⁵ M) (Shrivastava et al. 2012). However, the enzyme’s apparent maximum velocity (*V*_max app_) was approximately 28.75 times higher than *V*_max_ of L-asparaginase from *Cladosporium* sp. (*V*_max_ = 4.44 µmol mL⁻¹ min⁻¹) (Mohan and Manonmani 2013). However, the kinetic parameters for L-asparaginase from *C. echinulata* PA3S12MM are specific to enzyme partially purified and potential protein-protein interactions in the extract might influence the observed values. The low *K*_m app_ value (0.011 mmol L⁻¹) and the reduced glutaminase activity observed for *C. echinulata* L-asparaginase are promising biochemical indicators. These properties are essential prerequisites for enzymes intended for leukaemia treatment, as they suggest high efficiency in asparagine clearance and potentially fewer side effects. However, since this study focused on biochemical prospection, further research - including cytotoxicity assays in leukemic cell lines and immunological evaluations - is strictly necessary to validate its safety and efficacy for biomedical use.

## Conclusion

In conclusion, this study described the production and characterisation of a novel extracellular L-asparaginase from *Cunninghamella echinulata* PA3S12MM, a wild strain isolated from the Atlantic Forest. The enzyme exhibited exceptional thermotolerance, with optimal activity at 85 °C and remarkable stability at 95 °C, surpassing many previously reported fungal L-asparaginases. Furthermore, the successful use of chicken feathers as a low-cost nitrogen source demonstrates a sustainable approach for large-scale production. Given its high substrate affinity and thermal robustness, this enzyme represents a promising candidate for further investigation regarding its application in the food industry, particularly for acrylamide reduction, and as a candidate biopharmaceutical tool. While the current biochemical data provide a strong foundation, the transition to clinical or industrial applications will require future studies to assess its biocompatibility and performance in complex biological systems.

## Data Availability

The datasets generated during and/or analysed during the current study are available from the corresponding author on reasonable request.

## References

[CR1] Arumugam N, Thangavelu P (2022) Purification and anticancer activity of glutaminase and urease free intracellular L-asparaginase from *Chaetomium sp.* Protein Expr Purif 190:106006. 10.1016/j.pep.2021.106006 34742913 10.1016/j.pep.2021.106006

[CR2] Arumugam N, Shanmugam MK, Thangavelu P (2021) Purification and anticancer activity of glutaminase and urease-free L-asparaginase from novel endophyte *Chaetomium sp.* Biotechnol Appl Biochem 69:2161–2175. 10.1002/bab.2276 34694636 10.1002/bab.2276

[CR3] Asha S, Vidyavathi M (2009) Cunninghamella: a microbial model for drug metabolism studies—a review. Biotechnol Adv 27:16–29. 10.1016/j.biotechadv.2008.07.005 18775773 10.1016/j.biotechadv.2008.07.005

[CR4] Baruchel A, Brown P, Rizzari C, Silverman L, van der Sluis I, Wolthers BO, Schmiegelow K (2020) Increasing completion of asparaginase treatment in childhood acute lymphoblastic leukaemia (ALL): summary of an expert panel discussion. ESMO Open 5:e000977. 10.1136/esmoopen-2020-000977 32967920 10.1136/esmoopen-2020-000977 PMC7513670

[CR5] Bradford MM (1976) A rapid and sensitive method for the quantitation of microgram quantities of protein utilizing the principle of protein-dye binding. Anal Biochem 72:248–254 942051 10.1016/0003-2697(76)90527-3

[CR6] Cachumba JJM, Antunes FAF, Peres GFD, Brumano LP, Santos JCD, Silva SS (2016) Current applications and different approaches for microbial L-asparaginase production. Braz J Microbiol 47:77–85. 10.1016/j.bjm.2016.10.004 27866936 10.1016/j.bjm.2016.10.004 PMC5156506

[CR7] Cavalheiro GF, Costa AC, Garbin AP, Silva GA, Garcia NFL, Paz MF, Fonseca GG, Leite RSR (2023) Catalytic properties of amylases produced by *Cunninghamella echinulata* and *Rhizopus microsporus*. An Acad Bras Cienc 95:e20230187. 10.1590/0001-3765202320230187 37909570 10.1590/0001-3765202320230187

[CR8] Chow Y, Ting ASY (2015) Endophytic L-asparaginase-producing fungi from plants associated with anticancer properties. J Adv Res 6:869–876. 10.1016/j.jare.2014.07.005 26644924 10.1016/j.jare.2014.07.005 PMC4642164

[CR9] da Cunha MC, Aguilar JGS, de Melo RR, Nagamatsu ST, Ali F, de Castro RJS, Sato HH (2019) Fungal L-asparaginase: strategies for production and food applications. Food Res Int 126:108658. 10.1016/j.foodres.2019.108658 31732030 10.1016/j.foodres.2019.108658

[CR10] da Cunha MC, Aguilar JGS, de Melo RR, de Castro RJS, Sato HH (2022) L-asparaginase from Aspergillus oryzae spp.: effects of production process and biochemical parameters. Prep Biochem Biotechnol 52:253–263. 10.1080/10826068.2021.1931881 34110268 10.1080/10826068.2021.1931881

[CR11] Dumina M, Zhgun A (2023) Thermo-L-asparaginases: from the role in the viability of thermophiles and hyperthermophiles at high temperatures to a molecular understanding of their thermoactivity and thermostability. Int J Mol Sci 24:2674. 10.3390/ijms24032674 36768996 10.3390/ijms24032674 PMC9916696

[CR12] El-Gendy MMAA, Awad MF, El-Shenawy FS, El-Bondkly AMA (2021) Production, purification, characterization, antioxidant and antiproliferative activities of extracellular L-asparaginase produced by *Fusarium equiseti* AHMF4. Saudi J Biol Sci 28:2540–2548. 10.1016/j.sjbs.2021.01.058 33911966 10.1016/j.sjbs.2021.01.058 PMC8071902

[CR13] Gulati R, Saxena RK, Gupta R (1997) A rapid plate assay for screening L-asparaginase-producing microorganisms. Lett Appl Microbiol 24:23–26. 10.1046/j.1472-765X.1997.00331.x 9024001 10.1046/j.1472-765x.1997.00331.x

[CR14] Hong S, Lee Y, Khan AR, Ullah I, Lee C, Park CK (2014) Cloning, expression, and characterization of thermophilic L-asparaginase from *Thermococcus kodakarensis* KOD1. J Basic Microbiol 54:500–508. 10.1002/jobm.201300741 24442710 10.1002/jobm.201300741

[CR15] Imada A, Igarasi S, Nakahama K, Isono M (1973) Asparaginase and glutaminase activities of microorganisms. J Gen Microbiol 76:85–99 4723072 10.1099/00221287-76-1-85

[CR16] Jia R, Wan X, Geng X, Xue D, Xie Z, Chen C (2021) Microbial L-asparaginase for application in acrylamide mitigation from food: current research status and future perspectives. Microorganisms 9:1659. 10.3390/microorganisms9081659 34442737 10.3390/microorganisms9081659 PMC8400838

[CR17] Khalil NM, Rodríguez-Couto S, El-Ghany MNA (2021) Characterization of *Penicillium crustosum*L-asparaginase and its acrylamide alleviation efficiency in roasted coffee beans at non-cytotoxic levels. Arch Microbiol 203:2625–2637. 10.1007/s00203-021-02198-6 33709160 10.1007/s00203-021-02198-6

[CR18] Kim JK, Lee C, Lim SW, Adhikari A, Andring JT, McKenna R, Ghim CM, Kim CU (2020) Elucidating the role of metal ions in carbonic anhydrase catalysis. Nat Commun 11:4557. 10.1038/s41467-020-18425-5 32917908 10.1038/s41467-020-18425-5 PMC7486293

[CR19] Laemmli UK (1970) Cleavage of structural proteins during the assembly of the head of bacteriophage T4. Nature 227:680–685. 10.1038/227680a0 5432063 10.1038/227680a0

[CR20] Ma B, Huang HH, Chen XY, Sun YM, Lin LH, Zhong DF (2007) Biotransformation of metoprolol by the fungus *Cunninghamella blakesleeana*. Acta Pharmacol Sin 28:1067–1074. 10.1111/j.1745-7254.2007.00567.x 17588344 10.1111/j.1745-7254.2007.00567.x

[CR21] McIlvaine TC (1921) A buffer solution for colorimetric comparison. J Biol Chem 49:183–186. 10.1016/S0021-9258(18)86000-8

[CR22] Michaelis L, Menten ML (1913) Die Kinetik Der Invertinwirkung. Biochem Z 49:333–369

[CR23] Mohan Kumar NS, Manonmani HK (2013) Purification, characterization and kinetic properties of extracellular L-asparaginase produced by *Cladosporium sp.* World J Microbiol Biotechnol 29:577–587. 10.1007/s11274-012-1213-0 23180548 10.1007/s11274-012-1213-0

[CR24] Muneer F, Siddique MH, Azeem F, Rasul I, Muzammil S, Zubair M, Afzal M, Nadeem H (2020) Microbial L-asparaginase: purification, characterization and applications. Arch Microbiol 202:967–981. 10.1007/s00203-020-01814-1 32052094 10.1007/s00203-020-01814-1

[CR25] Parashiva J, Rameshgowda B, Madeva N, Raju B (2023) Response surface methodology based optimized production, purification, and characterization of L-asparaginase from *Fusarium foetens*. World J Microbiol Biotechnol 39:252. 10.1007/s11274-023-03610-0 37442849 10.1007/s11274-023-03684-3

[CR26] Ramos RCPDS, de Oliveira NS, Bianchini LF, Azevedo-Alanis LR, Pimentel IC, Hardy AMTG, Murata RM, Glassey J, Rosa EAR (2024) *Cunninghamella echinulata* DSM1905 biofilm-based L-asparaginase production in pneumatically driven bioreactors. PLoS One 19:e0308847. 10.1371/journal.pone.0308847 39302957 10.1371/journal.pone.0308847 PMC11414969

[CR27] Rasbold LM, Delai VM, da Cruz Kerber CM, Simões MR, Heinen PR, da Conceição Silva JL, de Cássia Garcia Simão R, Kadowaki MK, Maller A (2022) Production, immobilization and application of invertase from new wild strain *Cunninghamella echinulata* PA3S12MM. J Appl Microbiol 132:2832–2843. 10.1111/jam.15394 34850500 10.1111/jam.15394

[CR28] Shakambari G, Ashokkumar B, Varalakshmi P (2018) L-asparaginase: a promising biocatalyst for industrial and clinical applications. Biocatal Agric Biotechnol. 10.1016/j.bcab.2018.11.018

[CR29] Shrivastava A, Khan AA, Shrivastav A, Jain SK, Singhal PK (2012) Kinetic studies of L-asparaginase from *Penicillium digitatum*. Prep Biochem Biotechnol 42:574–581. 10.1080/10826068.2012.672943 23030468 10.1080/10826068.2012.672943

[CR30] Shrivastava A, Khan AA, Khurshid M, Kalam MA, Jain SK, Singhal PK (2016) Recent developments in L-asparaginase discovery and its potential as anticancer agent. Crit Rev Oncol Hematol 100:1–10. 10.1016/j.critrevonc.2015.01.002 25630663 10.1016/j.critrevonc.2015.01.002

[CR31] Thakur M, Lincoln L, Niyonzima FN, Sunil SM (2014) Isolation, purification and characterization of fungal extracellular L-asparaginase from *Mucor hiemalis*. J Biocatal Biotransform 2:1–9

[CR32] Trivedi M, Laurence J, Siahaan T (2009) The role of thiols and disulfides on protein stability. Curr Protein Pept Sci 10:614–625. 10.2174/138920309789630534 19538140 10.2174/138920309789630534 PMC3319691

[CR33] Vala AK, Sachaniya B, Dudhagara D, Panseriya HZ, Gosai H, Rawal R, Dave BP (2018) Characterization of L-asparaginase from marine-derived *Aspergillus niger* AKV-MKBU. Int J Biol Macromol 108:41–46. 10.1016/j.ijbiomac.2017.11.114 29175524 10.1016/j.ijbiomac.2017.11.114

[CR34] Wei R, Keang C, Song P, Su A, Ting Y (2024) Antioxidant properties and L-asparaginase activities of endophytic fungi from *Cymbidium orchids*. Folia Microbiol 69:713–722. 10.1007/s12223-023-01112-5 37995083 10.1007/s12223-023-01112-5

